# Direct micro-electric stimulation alters phenanthrene-degrading metabolic activities of *Pseudomonas* sp. strain DGYH-12 in modified bioelectrochemical system

**DOI:** 10.1007/s11356-019-05670-5

**Published:** 2019-09-02

**Authors:** Xingbiao Wang, Guilong Wan, Liuyang Shi, Xiaolong Gao, Xiaoxia Zhang, Xiaoguang Li, Jianfang Zhao, Beibei Sha, Zhiyong Huang

**Affiliations:** 1 Tianjin Institute of Industrial Biotechnology, Chinese Academy of Sciences, Tianjin, 300308 People’s Republic of China; 2 University of Chinese Academy of Sciences, Beijing, 100049 People’s Republic of China; 3 Binzhou Engineering Technology Research Center for High Salt Wastewater Treatment (chips) of Befar Group, Binzhou, Shandong Province 256602 People’s Republic of China

**Keywords:** Phenanthrene degradation, *Pseudomonas* sp. DGYH-12, Electrochemically assisted bioremediation, Phenanthrene metabolic pathways, Extracellular polymeric substance

## Abstract

Bioelectrochemical systems (BESs) have great potential for treating wastewater containing polycyclic aromatic hydrocarbons (PAHs); however, detailed data on cell physiological activities in PAH biodegradation pathways stimulated by BESs are still lacking. In this paper, a novel BES device was assembled to promote the growth of *Pseudomonas* sp. DGYH-12 in phenanthrene (PHE) degradation. The results showed that in the micro-electric field (0.2 V), cell growth rate and PHE degradation efficiency were 22% and 27.2% higher than biological control without electric stimulation (BC), respectively. The extracellular polymeric substance (EPS) concentration in BES (39.38 mg L^−1^) was higher than control (33.36 mg L^−1^); moreover, the membrane permeability and ATPase activities were also enhanced and there existing phthalic acid and salicylic acid metabolic pathways in the strain. The degradation genes *nahAc*, *pcaH*, and *xylE* expression levels were upregulated by micro-electric stimulation. This is the first study to analyze the physiological and metabolic effect of micro-electric stimulation on a PHE-degrading strain in detail and systematically.

**Electronic supplementary material:**

The online version of this article (10.1007/s11356-019-05670-5) contains supplementary material, which is available to authorized users.

## Introduction

Polycyclic aromatic hydrocarbons (PAHs), a wide class of aromatic hydrocarbons with more than one benzene ring, are ubiquitous contaminants in wastewater derived from both natural and anthropogenic activities (Ghosal et al. 2010). Phenanthrene (PHE), one of the most widespread PAHs with three-fused rings in an angular position, is considered to be a major pollutant in soil and water (Masakorala et al. 2013). PHE is usually categorized as a typical compound for PAH biodegradation studies (Gao et al. 2013; Isaac et al. 2013). It is worth noting that PAHs have prolonged persistence, recalcitrance, mutagenic potential, and carcinogenic properties (Lin et al. 2014). Owing to its abundance, toxicity, and intrinsic chemical stability in the environment, controlling PAHs has been a research hotspot for decades worldwide. For example, the United States Environmental Protection Agency (EPA) categorizes 16 PAHs as primary pollutants (Hussein and Mona 2016). Hence, it is of great necessity to treat PAH-containing wastewater and reduce or eliminate environmental hazards.

PHE as a kind of PAHs exists in the natural environment widely, mainly in coal tar, coking wastewater, pharmaceutical wastewater, fuel wastewater, resin manufacturing, and other industrial pollutants (Edlund and Jansson 2008; Gao et al. 2013; Roy et al. 2012). PHE can enter to humans and animals’ body through inhalation, ingestion, and skin contact, causing potential health problems (Gao et al. 2013; Roy et al. 2012). The environment polluted by phenanthrene is often accompanied by other PAHs and heterocyclic aromatic hydrocarbons (Lin et al. 2014). The migration of water pollution also causes soil pollution, groundwater pollution, and human malignant diseases (Muratova et al. 2014; Masakorala et al. 2013). When the total content of PAHs in industrial wastewater is 20–27 μg/L, the phenanthrene concentration is 6–17 μg/L. At the same time, the biological toxicity of phenanthrene easily leads to the instability of microbial treatment system, which further increases the difficulty of degradation of phenanthrene (Edlund and Jansson 2008; Pan et al. 2010; Ghosal et al. 2010; Zhao et al. 2008).

To date, a range of physical, chemical, and biological methods involved in detoxifying PAHs from wastewater have been developed. Wlodarczyk-Makula utilized ultraviolet rays to decrease the concentration of PAHs in industrial wastewater by more than 50% (Wlodarczyk-Makula et al. 2016). Turek attempted to remove PAHs using dihydrogen dioxide in the presence of metal catalysts, such as cobalt, platinum, and titanium; the increase in the amount of dihydrogen dioxide increased the oxidation efficiency by 31% (Turek et al. 2016). Although these physical and chemical methods can detoxify PAHs, unavoidable issues are that usually cause secondary pollution to the environment or have high treatment costs. Based on the above research background, microbial degradation has been a focus of research due to it being more cost effective and environmentally acceptable for detoxifying PAH contaminants (Masakorala et al. 2013). Nonetheless, the low biodegradation efficiency critically hinders the industrial scale application of the microbial method due to the refractory property of PAHs. Therefore, a prerequisite is finding applicable methods to enhance activity and PAHs’ toxicity resistance, to promote and extend their application in PAH bioremediation.

One effective way to enhance the microbial biodegradation efficiency is applying micro-electric stimulation in a bioelectrochemical system (BES) (Nuerla Ailijiang et al., 2016; Liu et al. 2015), which combines micro-electric fields and biological processes (Zhang et al. 2012; Gambino et al. 2017; Mousset et al. 2016) in a device. In the last few decades, various BESs have been applied to treat organic pollutants, such as 2-fluoroaniline (Zhang et al. 2014), azo dye (Kong et al. 2015), and phenol (Nuerla Ailijiang et al., 2016). In BESs, the pollutants are biodegraded with a higher efficiency by microbial cells via micro-electric stimulation.

Although the application of BESs in wastewater treatment has gained extensive attention, studies on the relationships between microorganisms and BESs have mainly focused on system performance (Thrash and Coates 2008; Zhang et al. 2014). To our knowledge, few studies can be found that explain how the stimulation of micro-electric fields can improve wastewater treatment performance with microorganisms. Herein, a systematical study on the mechanisms between microorganisms and micro-electric field during the bioelectrochemical degradation of recalcitrant pollutants was carried out.

In this study, four newly designed BESs were constructed for strain DGYH-12 to degrade PHE. The reactor performance was assessed in terms of the PHE degradation efficiency and cell growth rate of DGYH-12. Furthermore, to understand how micro-electric stimulation affects the performance of DGYH-12, several aspects were brought into the analysis, including morphological changes, EPS productions, ATPase activities, degradation pathways, membrane permeability, and PHE-degrading gene expressions. This is the first study that analyzes the effect of micro-electric stimulation on a PHE-degrading strain in details. This work is expected to be a useful supplement in the development of BESs and offer a preliminary orientation for using BESs in the treatment of PHE-contaminated wastewater.

## Materials and methods

### Isolation and identification of PHE-degrading strain

Crude oil polluted soil was collected from the Dagang oilfields, Tianjin, northeast China. A modified “enrichment-screening-rescreening” strategy was utilized to obtain the desired PHE-degrading strains (Zhao et al. 2008). In this study, all reagents were of analytical grade. The mineral salt medium (MSM) was composed of the following (L^−1^): Na_2_HPO_4_·12H_2_O 8.5 g, KH_2_PO_4_ 3.0 g, NaCl 0.5 g, NH_4_Cl 1.0 g, MgSO_4_·7H_2_O 0.5 g, and CaCl_2_ 14.7 mg (Zhao et al. 2008). MSM also contained the following trace elements (L^−1^): ZnSO_4_ 0.4 mg, MnSO_4_ 4.0 mg, FeSO_4_ 2.0 mg, CuSO_4_ 0.4 mg, CoCl_2_ 0.4 mg, KI 1.0 mg, H_3_BO_3_ 5.0 mg, NaMoO_4_·2H_2_O 1.0 mg, and FeCl_3_·6H_2_O 2.0 mg. The pH of the medium was adjusted to 7.0 (Zhao et al. 2008). The solid Luria Bertani (LB) medium was according to the reference (Masakorala et al. 2013). First, 2 g soil was inoculated in 50 mL sterilized MSM medium and incubated aerobically at 30 °C while shaking at 180 rpm in the dark for 3 h. After 1 h standing, 2 mL of the supernatant was transferred to 100 mL sterilized MSM with the addition of 10 mg PHE as the sole carbon and energy source. After incubating in shake flasks at 30 °C for 7 days, 1 mL of enriched aqueous culture was transferred to 100 mL of fresh MSM containing 10 mg of PHE weekly and incubated in the same conditions described above. This consecutive enrichment process was repeated four times. Next, 100 μL of enriched cells was harvested and inoculated on solid LB plates. After 2 days of incubation, the colonies were isolated and transferred into 24-well culture plates containing 5 mL of MSM and 100 mg L^−1^ PHE in each well. After 7 days of incubation, potential PHE-degrading cells were isolated from 24-well culture plates. The harvested cells were streaked on the solid LB plates and purified 3 times to ensure the strain’s purity, respectively.

Identification was carried out through phylogenetic analysis based on the sequencing results of 16S rDNA. The morphological, physiological, and phylogenetic characteristics of the strain were analyzed according to procedures that have been described previously (Dong et al. 2015).

Genomic DNA was extracted from purified strains and amplified by PCR using the following universal primers: F_27_ (5′-AGAGTTTGATCCTGCTCAG-3′) and R_1492_ (5′-GGTTACCTTGTTACGCTT-3′) (Masakorala et al. 2013). The 16S rDNA sequences were checked manually and underwent phylogenetic analysis. The sequence similarities were analyzed from the database (www.ncbi.nln.nih.gov) and sequence alignment was performed through software Clustal X (version 2.0).

### Reactor configuration

**Fig. 1:**
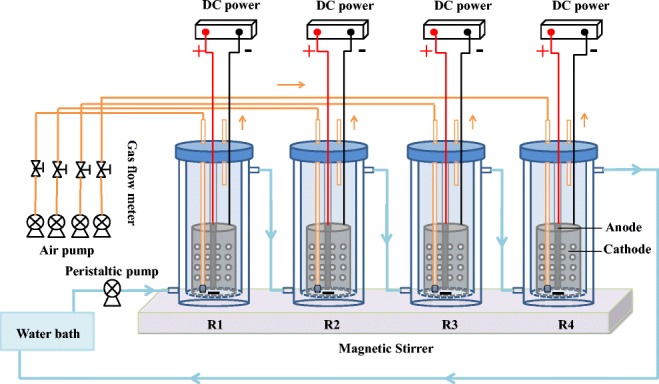
Conceptual design of a novel bioelectrochemical system (BES). R1, R2, R3, and R4 are the experimental group, simply electrochemical control, biological control without an electric field, and control check (CK), respectively

The concept design of four new styles of BESs, which is shown in Fig. 1, includes five parts: the reactor, the magnetic mixing system, the aerating system, the heating system, and the micro-electric field generating system. The effects of electrical stimulation mainly depend on the electrode type, electric intensity, microorganism species, etc. (Nuerla Ailijiang et al., 2016). To increase the uniformity of the electric stimulation, the surrounding deployment of electrodes was adopted.

As the original design shown in Fig. 1, a cylindrical graphite electrode (5 cm, 6 cm, and 7.5 cm in inner diameter, outer diameter, and height, respectively) with uniform holes (0.5 cm in radius and 2 cm in hole pitch) on the surface and a rod graphite electrode (1 cm in inner diameter and 7.5 cm in length) was fixed into a cylindrical glass beaker (6.5 cm in inner diameter and 20 cm in height) with a total active liquid volume of 200 mL. The rod graphite electrode acted as an anode and was fixed in the middle of the treatment, and the cylindrical graphite electrode with many small holes served as a cathode. The top of each electrode was approximately one-third of the way down the reactor, and the distance between electrodes was 2 cm. A direct current (DC) power device was connected with the BES to supply continuous external power between the anode and cathode. An aeration device was fixed in the center of the reactor, and a magnetic stirring system and a water bath heating system were assembled to improve the homogeneity of the medium and achieve temperature control. Three other reactors were utilized as controlled experiments, including an electrochemical control without the PHE-degrading strain (R2), a biological control without the electric field (R3), and a CK (R4), in which the other settings and culture conditions are the same as in R1.

### Optimization of direct micro-electric fields

Considering that only suitable micro-electric stimulation could have a positive effect on bacteria, the optimization of the micro-electric field response to the PHE-degrading strain was carried out as follows. Three reactors were exposed to micro-electric stimulation, and the last one was set as a biological control with microbial inoculation, but no electric field. The reactor contained 200 mL of MSM medium and 100 mg L^−1^ PHE. After being seeded with 20 mL of 2-day-old preculture cells, the four reactors were operated at 30 °C ± 1 °C for 7 days. To quantitatively evaluate the effects of micro-electric stimulation on the viability of PHE-degrading strain, the cell density and PHE degradation rate were assessed periodically.

### Extraction and analysis of PHE

PHE was extracted with equal volume of *n*-hexane 3 times. Then, the extracts were mixed and dried by anhydrous sodium sulfate and concentrated to dryness. They were then re-dissolved in 1 mL of acetone to test the rate of PHE degradation. The concentrated PHE was analyzed using a high-performance liquid chromatograph (LC-20AD, SHIMADZU, Japan) according to the reported method (Isaac et al. 2013).

A fixed wavelength UV detector at 254 nm and a SinoChrom ODS-BP column (250 mm × 4.6 mm) were set in the LC-MS. The column temperature was maintained at 30 °C. The mobile phase was composed of 10% water and 90% methanol, and the flow rate was 0.5 mL min^−1^. The injection volume was 10.0 μL. PHE was detected at 14 min after sample injection. The PHE concentration was quantified using the external standard method (Isaac et al. 2013).

To indicate the effects of micro-electric stimulation on strain DGYH-12, the following physicochemical properties were also determined during the treatment processes.

### Morphological characteristics and cell density analysis

To explain how micro-electric stimulation influenced the PHE-degrading strain, the effects of micro-electric stimulation on the morphology changes of the PHE-degrading strain were analyzed.

After sampling, 1 mL of fermentation broth was immediately fixed with glutaraldehyde (2.5% aqueous solution) for 24 h at 4 °C and processed through an ethanol dehydration series (i.e., 30, 50, 70, 85, 95, and 100%, *v*/*v*, ethanol, 0.5 h each treatment). Before examination by scanning electron microscopy (SU 8010, Hitachi, Japan) and transmission electron microscopy (HT 7700, Hitachi, Japan), the cell samples were treated by critical point drying to maintain the original morphology.

On account of the insolubility of PHE in a water-based culture, the optical density (OD) of cells will be affected by the non-uniformity of PHE, leading to inaccuracy in the cell density. Therefore, the cell density of DGYH-12 was checked via the microscopic count method daily instead of the spectrophotometric method.

### EPS extraction and chemical analysis

To investigate the effects of micro-electric stimulation on the extracellular polymeric substance (EPS) secretion in the PHE degradation process, EPS was extracted according to the reported methods with small modifications (Pan et al. 2010).

Cell suspension was centrifuged at 4300*g* for 10 min at 4 °C to remove the supernatant culture medium. The residues were suspended in deionized water and centrifuged at 20,000*g* for 20 min at 4 °C to separate the EPS. The supernatant was filtered through a 0.22-μm membrane, and then, the residue was harvested as raw EPS. The raw EPS was dialyzed through dialysis membrane (3500 Da) in deionized water at 4 °C for 24 h, and purified EPS was obtained and stored at 4 °C until use.

Protein in the EPS was determined by the modified Lowry method (Hanne et al. 2018) using bovine serum albumin (BSA) as the standard reference, whereas polysaccharides in the EPS were measured by the phenol-sulfuric acid method with glucose as the standard reference (Dubois et al. 1956). DNA in the EPS was determined by the diphenylamine colorimetric method with 2-deoxy-D-ribose as the standard reference (Zhao et al. 2013).

### ATPase activity and cell membrane permeability analysis

The ATPase activity was used as the main index of the total metabolic activity (Nuerla et al. 2016). In this study, the intracellular ATPase activity of strain DGYH-12 was analyzed every 2 days using an ATPase analysis kit (Solarbio, China). On account of changes in the electrochemical membrane potential, which affects the membrane transportation and process energy transfer, the low electric field could induce changes in the concentration of ATP in *Saccharomyces cerevisiae* (Lustrato et al. 2006). The total ATP content increased during the low-intensity electric current stimulation in the catabolic metabolism of *Aspergillus niger* compared with the control without an electric current (Velasco-Alvarez et al. 2011). In addition, the micro-electric stimulation boosted the metabolic activity of microorganism probably at the mitochondrial level, as proposed by Vajrala et al. which demonstrates that a low electric current can cause mitochondrial modifications (Vajrala et al. 2008).

To analyze the effect of micro-electric stimulation on the membrane permeability of strain DGYH-12, the cell membrane permeability was measured based on the amount of extracellular protein after centrifugation at 5000 rpm for 20 min. These protein extractions were determined according to the modified Lowry method (Hanne et al. 2018).

### Extraction and identification of PHE-degrading metabolites

To understand the metabolic processes of PHE degradation in the BES to analyze the effects of micro-electric stimulation on PHE degradation-related gene expression, the metabolite extraction and identification were carried out as follows.

After incubation for 7 days, the cultures were centrifuged at 8000 rpm for 10 min, and the supernatant was acidified to pH 2.0 with 12 N HCl and extracted with equal volumes of ethyl acetate 3 times. The combined organic layer was re-extracted with 10 mM aqueous sodium hydroxide. The remaining organic phase was dried over anhydrous sodium sulfate and concentrated up to dryness using a pressure blowing concentrator (neutral fraction). The pH of the aqueous sodium hydroxide extraction was altered to 2.0 as described above and then extracted with equal volume of ethyl acetate. The extraction was dried over anhydrous sodium sulfate and concentrated up to dryness using a pressure blowing concentrator (acidic fraction) (Gao et al. 2013).

Metabolites in both the neutral fraction and acidic fraction were derivatized with bis-trimethylsilyl-trifluoroacetamide (BSTFA) and characterized by gas chromatography mass spectrometry (GC-MS). GC-MS analysis was performed on a gas chromatograph (7890A, Agilent Technologies, USA) mass (7200, Agilent Technologies, USA) equipped with a HP-5MS column (30 m, 0.25 μm). A 1.0-μl sample was injected in splitless mode with an autosampler (7693, Agilent Technologies, USA). The purge value was activated 3 min after the sample injection; helium was used as a carrier gas with a flow rate 2 mL min^−1^. The column temperature started at 120 °C for 2 min and then programmed to 280 °C at a rate of 2 °C min^−1^, and was held at 280 °C for 10 min. The injector and transfer line temperatures were set to 270 and 280 °C, respectively. The mass spectrometer was operated in electron impact mode (Gao et al. 2013).

### Primer design, DNA extraction, RNA extraction, cDNA formation, and gene expression quantification

To investigate the effects of micro-electric stimulation on the PHE degradation-related gene expression, a quantitative real-time PCR technique was adopted to estimate the copy number of genes. The total DNA of strain DGYH-12 was extracted using the TIANamp Bacteria DNA KIT (TIANGEN, China) according to the operating specifications. The DNA concentration was determined by the NanoDrop Spectrophotometer (ND-1000, Thermo Fisher Scientific, USA). The genes *nahAc*, *pcaH*, and *xylE* were cloned in total DNA using the primers shown in Table 3. The gene *nahAc* encoded a large subunit of the naphthalene dioxygenase system (a three-component class III oxygenase), which was associated with the initial catalysis step during the PHE biodegradation process (Isaac et al. 2015). The gene *pcaH* encoded the beta subunits of protocatechhuate-3,4-dioxygenase, which was expressed in the phthalic acid metabolic pathway (Badejo et al. 2013). The gene *xylE* encoded the catechol-1, 2-dioxygenase, which was related to the salicylic acid metabolic pathway (Edlund and Jansson 2008).

The total RNA of strain DGYH-12 was extracted by the TIANamp Bacteria total RNA KIT (TIANGEN, China). cDNA reverse transcription was completed by RevertAid First Strand cDNA Synthesis Kit (Thermo scientific, USA). Quantitative real-time PCR reactions for amplifying the *nahAc*, *pcaH*, *xylE*, and 16S rRNA genes were performed using the SuperReal PreMix Plus (SYBR Green) kit (TIANGEN, China). The internal housekeeping 16S rRNA gene was amplified together with *nahAc*, *pcaH*, and *xylE* gene expression to serve as a reference point to evaluate changes in the target gene expression (Table 1).

**Table 1:** Primers used for 16S rRNA, *nahAc*, *pcaH*, and *xylE* gene amplification

Primer	Sequence (5′ → 3′)	Amplicon size (bp)	Target gene	Reference
1F	AGAGTTTGATCCTGCTCAG	1321	16s rRNA	Masakorala et al. (2013)
1R	GGTTACCTTGTTACGCTT			
2F	GAGATGCATACCACGTKGGTTGGA	1125	*nahAc*	Cebron et al. (2008)
2R	CGGCGCCGACAAYTTYGTNGG			
3F	GTTGAGACTGGCGAACGGTA	505	*pcaH*	Badejo et al. (2013)
3R	AATGTTCAGCAAACGCGAGG			
4F	TGGAGCGAGGTGGACAA	796	*xylE*	This study*
4R	AGGCTGAGGAACTGGGAG			

*Primers designed for the *xylE* gene were based on the *Pseudomonas plecoglossicida* strain NyZ12 with GenBank accession no. EF544606.2

Quantitative real-time PCR was employed on the Roche real-time thermal cycler (Roche LightCycler®96, USA). The PCR volume was 20 μL, containing 0.4 μM for each primer (Table 4). Each PCR included a no-template control with water instead of cDNA as well as a RT negative control for each gene. Triplicate measurements were performed for all reactions. The results were analyzed utilizing the critical threshold (ΔC_T_) method (Table 2).

**Table 2:** Primers used in RT-PCR in this study

Primer	Sequence (5′ → 3′)	Amplicon size (bp)	Target gene	Reference
1F	CCGACAGAATAAGCACCG	216	16s rRNA	This study*
1R	CTACGCATTTCACCGCTA			
2F	GATCCTTACCACCCGCTTCC	150	*nahAc*	This study*
2R	CCTTGATTGATTCGTGACCTCC			
3F	CAGATGCCGGACAACAA	140	*pcaH*	This study*
3R	GAAGACCCGTCTCACTATGA			
4F	CTTCTCCCTTTGCGACTG	150	*xylE*	This study*
4R	CGACCTGATTCCCGACTT			

*All primers were designed by Primer 5.0 software according to the sequences of the target gene in strain DGYH-12

### Data analysis

All of the figures in this paper were plotted using Origin Pro8.0 software. It is of great significance to note that all of the treatments and controls were performed in triplicate, and the average was calculated to ensure the accuracy of the results presented in this study. The significant difference between various data was analyzed and marked with normal letters; the different normal letters on the bar indicate significant difference among settings at 0.05 level according to the Duncan test.

## Results and discussion

### Isolation and identification of PHE-degrading strains

A modified enrichment-screening-rescreening strategy was adopted to isolate PHE-degrading strains. Twenty bacterial strains were isolated and purified. The growth characteristics of each isolate in the MSM medium, including 100 mg L^−1^ PHE, were tested. Among these isolates, strain DGYH-12 showed the highest PHE-degrading activity (3 days, 63%). It formed smooth, transparent, and wet colonies, which were light yellow and circular with a diameter of 1 to 1.5 mm. This strain was Gram-negative and had rod-like cells that grew aerobically. Furthermore, a physiological study showed that strain DGYH-12 could use urease, lysine decarboxylase, and ornithine decarboxylase, whereas it showed negative utilization in lactose, indole, cellobiose, xylose, melibiose, rhamnose, saccharose, raffinose, ONPG, malonate, gelatin, inositol, sorbitol, mannitol, amygdalin, esculoside, melezitose, xylitol, and salicin. The closest reference strain (99% identity) was the *Pseudomonas plecoglossicida* strain Ny Z12 (GenBank accession no. EF544606) through a comparison of the 16S rRNA gene sequence in the NCBI database. The morphological, physiological, and phylogenetic properties supported that strain DGYH-12 belonged to the species of *Pseudomonas plecoglossicida*.

### Optimization of direct micro-electric field

**Fig. 2:**
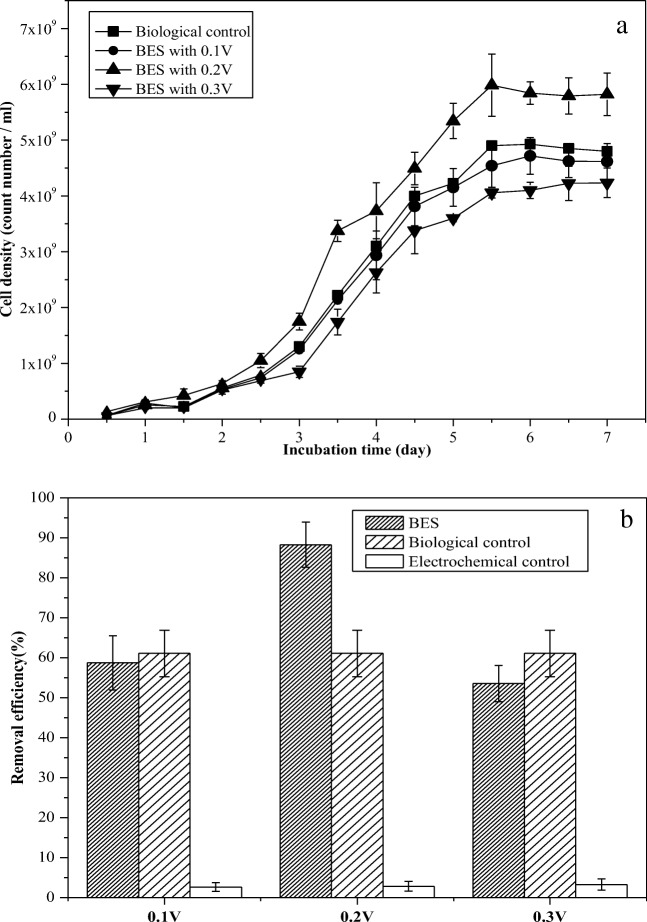
Effects of the various (0.1–0.3 V) electric fields on the growth activity (**a**) and degradation efficiency (**b**) of PHE-degrading bacteria *Pseudomonas* sp*.* DGYH-12. In the figure **b**, the data of BES at 0.2 V was compared with other data of BES, BC, and EC of different voltage settings; the significant differences were marked with normal letters; the different normal letters on the bar indicate significant difference among settings at 0.05 level according to the Duncan test

To test the micro-electric stimulation effects, the growth curve of strain DGYH-12 and the PHE degradation were measured (Fig. 2).

The cell growth process of strain DGYH-12 in the BES under various voltages from 0.1 to 0.3 V is shown in Fig. 2a. On day 7, the cell densities of strain DGYH-12 with voltages of 0.1 V, 0.2 V, and 0.3 V and the biological control were 4.6 × 10^9^, 5.82 × 10^9^, 4.23 × 10^9^, and 4.80 × 10^9^ mL^−1^, respectively. The cell densities under 0.1 V, 0.2 V, and 0.3 V were, respectively, 0.96, 1.21, and 0.88 times greater than that of the biological control. Strain DGYH-12 showed the highest cell density under 0.2 V of electric stimulation, which meant that 0.2 V of electric stimulation could effectively accelerate the cell growth of strain DGYH-12; 0.1 V of electric stimulation had little effect on the viability of strain DGYH-12, whereas 0.3 V significantly inhibited the growth of strain DGYH-12. Additional experiments also showed that the cell growth decreased dramatically if the voltage was higher than 0.3 V (data not shown).

The performance of strain DGYH-12 in terms of the PHE degradation activity is shown in Fig. 2b. On day 7, the PHE degradation rates in the BES under 0.1 V, 0.2 V, 0.3 V, and the biological control were 58.74 ± 6.77%, 88.27 ± 5.66%, 53.54 ± 4.53%, and 61.07 ± 5.80%, respectively. By contrast, the PHE degradation rates in the electrochemical control with 0.1 V, 0.2 V, and 0.3 V were 2.65 ± 1.10%, 2.85 ± 1.20%, and 3.24 ± 1.40%, separately. The PHE degradation rates under 0.1 V, 0.2 V, and 0.3 V were, respectively, 0.96, 1.45, and 0.87 times greater than that in the biological control. It can be seen from the data that 0.2 V micro-electric stimulation showed the best performance in promoting PHE degradation, showing the same trend as the cell growth progress in the BES, whereas 0.1 V micro-electric stimulation had a small effect on PHE degradation rate. By contrast, compared with biological control, 0.3 V of micro-electric stimulation decreased the PHE degradation rate by 17.53%. Furthermore, the electrochemical control results indicated that micro-electric stimulation without microbial effects indicated no PHE degradation influence.

Figure 2 reveals that the optimal micro-electric field could enhance the microorganisms PHE degradation performance, whereas excessive stress from electric fields caused negative effects on the activity of microbes, even leading to their death (Mena et al. 2014; Huang et al. 2014; Wei et al. 2011; Diao et al. 2004). This inactivation of microorganisms is due to thermal effects or the toxic substances generated by reactions at the electrodes, such as soluble metallic ions and free chlorine (Velasco-Alvarez et al. 2011).

The improvement in the PHE degradation rate can be attributed to the increased growth rate of strain DGYH-12 via suitable micro-electric stimulation. From the above results, it can be concluded that the 0.2 V electric field was the optimal voltage for stimulating strain DGYH-12 to degrade PHE in the BESs. Consequently, an optimum micro-electric field existed, which is in agreement with previous reports (Nuerla et al. 2016), demonstrating that the optimum electric field increased phenol biodegradation in the BESs and that the optimum micro-electric stimulation can effectively increase the growth rate and PHE degradation activity of strain DGYH-12.

### Morphological changes, EPS production characteristics, and cellular membrane permeability

One important point was that the specific biochemical mechanisms of the interactions between microorganisms and micro-electric stimulation are still unclear (Thrash and Coates 2008). To understand how micro-electric stimulation influenced the cells morphologically, the effect of micro-electric stimulation on the morphological changes of strain DGYH-12 was observed through scanning electron microscopy (SEM) and transmission electron microscopy (TEM).

**Fig. 3:**
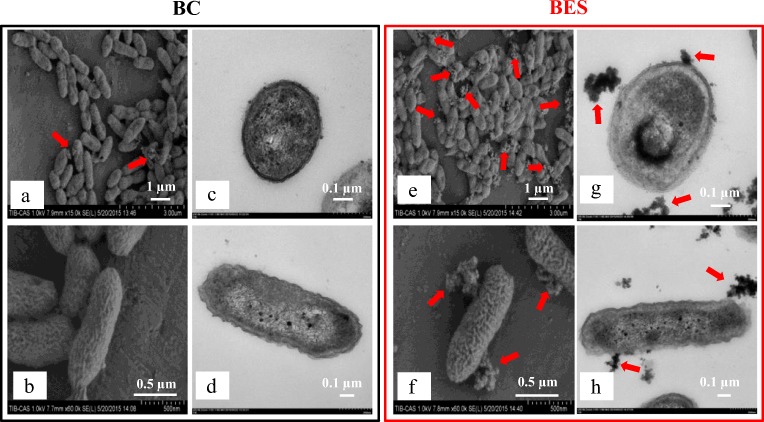
Morphological changes of *Pseudomonas* sp*.* DGYH-12 in the BES (marked in red rectangle) and the biological control (BC) reactors (marked in black rectangle) at mid-logarithmic phase. **a**, **b**, **e**, and **f** are the images of SEM; **c**, **d**, **g**, and **h** are the images of TEM

Figure 3 shows the surface morphology of strain DGYH-12 in the BES with 0.2 V of electric stimulation as well as cells in the biological control without electric stimulation. From Fig. 3, compared with the biological control (Fig. 3a), the microbial community structure in the BES (Fig. 3e) was denser. Additionally, a mass of excreted EPS was observed accumulated on the surfaces of strain DGYH-12 in the BES, which is marked by red arrows in Fig. 3. In SEM (Fig. 3b, f) and TEM images of a single cell, it was further suggested that the surface of cells had more EPS in the BES, which indicated that micro-electric stimulation could stimulate strain DGYH-12 to secrete more EPS.

**Fig. 4:**
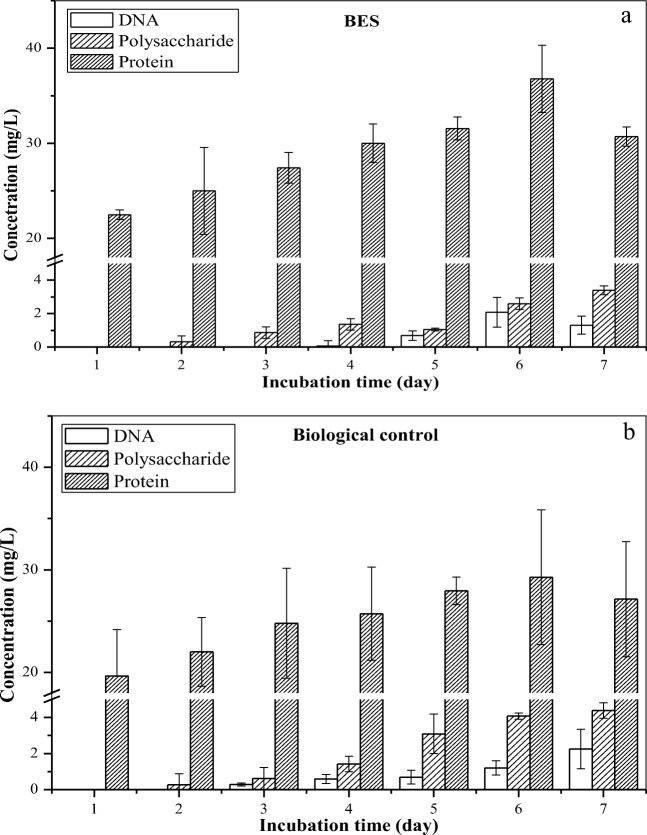
Extracellular polymeric substances of *Pseudomonas* sp*.* DGYH-12 in the BES (**a**) and BC (**b**). The EPS mainly consisted of proteins and polysaccharides. The DNA, polysaccharide, and protein data were compared with different incubation times, respectively. The significant differences were marked with normal letters; the different normal letters on the bar indicate significant difference among settings at 0.05 level according to the Duncan test. The different letters in DNA, polysaccharide, and protein data have no comparative relationship with each other

To further analyze the results obtained, as shown in Fig. 3, EPS was extracted from the BESs for quantitative analysis. Proteins and polysaccharides proved to be major biochemical components of the EPS (Zhang et al. 2015). To avoid the interference of intracellular substances in EPS analysis, DNA in the EPS was measured as an indicator of cell disruption caused by the extraction treatment of the EPS. Hence, the concentrations of proteins, polysaccharides, and DNA in the EPS were measured to analyze the effects of micro-electric stimulation on the EPS secretion of strain DGYH-12 (Fig. 4). From day 1 to day 6, the production of proteins in the BES (Fig. 4a) and biological control (Fig. 4b) increased from 22.50 and 19.64 to 36.79 and 29.29 mg L^−1^ and then decreased to 30.71 and 27.14 mg L^−1^ after incubation for 7 days, respectively. From day 1 to day 7, the concentrations of polysaccharides in the BES and biological control increased steadily from 0 to 3.40 and 4.38 mg L^−1^, respectively. The concentrations of DNA were relatively low, less than 2.09 and 2.25 mg L^−1^ in the BES and biological control, respectively. This phenomenon indicated that the extraction treatment did not cause cell disruption.

As shown in Fig. 4, the protein contents increased with the incubation time. The content peak of EPS, including the proteins and polysaccharides in the BES, was 39.38 mg L^−1^. In contrast, that in the biological control was 33.36 mg L^−1^. The proteins were upregulated in the BES compared with the biological control.

Interestingly, Fig. 4 indicates that strain DGYH-12 synthesized more proteins but less polysaccharides with PHE as the sole carbon source. According to previous research, this is a common trait of most bacteria during hydrophobic wastewater treatment (Zhang et al. 2011), which is involved in the regulation of the transport of organic pollutants across a bacterial cell membrane and involves a very complex metabolic pathway (Zhang et al. 2015).

One major factor restricting the bioremediation of PAHs in wastewater was the poor bioavailability for microbial cells (Zhang et al. 2011). Studies have proven that biofilms can enhance the solubility and bioavailability of poorly soluble PAHs for subsequent biodegradation (Zhang et al. 2015; Pan et al. 2010; Zhang et al. 2011). In biofilms, EPS showed significant correlation with PAH biodegradation, which could enhance cell adhesion, biofilm formation, cell transport, and the adsorption of hydrophobic compounds (Zhang et al. 2011). In addition, EPS was also reported to have hydrophobic moieties, which have strong affinity for hydrophobic toxic pollutants, such as PAHs (Pan et al. 2010). These results indicated that micro-electric stimulation indeed induced strain DGYH-12 to produce more EPS, which was beneficial to forming a complex and compact biofilm.

As displayed in Table 3, the protein concentration in the upper layer was detected to evaluate the cellular membrane permeability. In the BES and biological control, from 0 to 6 days, the protein concentration increased from 6.44 ± 0.54 mg L^−1^ to 20.36 ± 4.30 mg L^−1^ and 19.68 ± 3.16 mg L^−1^, respectively, and then decreased to 6.07 ± 0.54 and 4.64 ± 0.51 mg L^−1^ on 8 days, respectively. These results showed that the cellular membrane permeability gradually increased from 0 to 6 days and then decreased on 8 days in both the BES and biological control. By comparison, between the BES and biological control, cellular membrane permeability was slight higher in the BES than in the biological control. Micro-electric stimulation had a positive effect on the cellular membrane permeability, promoting strain DGYH-12 to synthesize more EPS, and the bioavailability of poorly soluble PHE could be enhanced. As a consequence, the growth and degradation activities of the strain were improved by micro-electric stimulation.

**Table 3:** Protein concentration in the upper layer of strain DGYH-12 in solution at different incubation times

Incubation time (day)	Protein concentration in the upper layer (mg L^−1^)	
	BES	BC
0	6.44 ± 0.54	6.44 ± 0.54
2	9.74 ± 4.34	8.96 ± 1.96
4	10.51 ± 1.82	8.80 ± 2.25
6	20.36 ± 4.30	19.68 ± 3.16
8	6.07 ± 2.53	4.64 ± 0.51

Previous research demonstrated that electric stimulation can change the properties of a cell membrane and a moderate direct electric field can increase transmembrane conductivity as well as the diffusive permeability of nutrients, surfactants, and autoinducers (Loghavi et al. 2008). Other research also reported that the application of electric fields can influence the orientation of membrane components, such as lipids (Shi et al. 2008; Wick et al. 2004), and this effect may result in irreversible permeabilization of the membrane (Wei et al. 2011).

All of the research described above supports the conclusion that the cellular membrane permeability could be increased with the application of micro-electric stimulation, which can promote microbial cells to secrete more EPS to protect themselves from electric stimulation. Because EPS can increase the bioavailability of PHE, the PHE degradation efficiency and growth rate of strain DGYH-12 could be increased by 0.2 V micro-electric stimulation. This was the first study to illustrate that micro-electric stimulation has a positive effect on the EPS production of a PHE-degrading strain.

### The ATPase activity

The ATPase activity could be used as a bioindicator to evaluate the metabolic activity of microorganisms (Velasco-Alvarez et al. 2011). In this study, the total ATPase activity of strain DGYH-12 was measured to understand the effects of micro-electric stimulation on the metabolic activity of strain DGYH-12. On the whole, the ATPase activity of strain DGYH-12 gradually increased over the incubation time of 6 days and then declined significantly from day 6 to day 8 (Table 4), in line with the variation trend of the cellular membrane permeability (Table 3). The ATPase activity of strain DGYH-12 in the BES was 2.66, 1.35, 1.22, and 1.35 times higher than the biological control on days 2, 4, 6, and 8, respectively.

**Table 4:** ATP enzyme activity of *Pseudomonas* sp. strain DGYH-12 in the BES and BC at different incubation times

Incubation time (day)	ATPase activity (U)	
	BES	BC
0	–	–
2	1.76 ± 0.62	0.66 ± 0.16
4	5.61 ± 2.64	4.14 ± 1.67
6	17.27 ± 1.76	14.10 ± 9.38
8	7.70 ± 2.67	5.69 ± 4.35

The above research illustrated that the ATPase activity of strain DGYH-12 can be improved by micro-electric stimulation, which is one reason the micro-electric field can improve the growth and degradation activity of strain DGYH-12.

### Metabolite identification and novel metabolic pathway deduction of PHE

To understand the biodegradation process of DGYH-12, PHE degradation metabolites were identified.

Previous studies have proven that the biodegradation mechanism of PHE mainly had two pathways, a phthalic acid pathway and a salicylic acid pathway (Gao et al. 2013; Muratova et al. 2014; Roy et al. 2012). Interestingly, in this research, a potential phthalic acid degradation pathway and a salicylic acid degradation pathway coexisted in PHE degradation of strain DGYH-12.

**Fig. 5:**
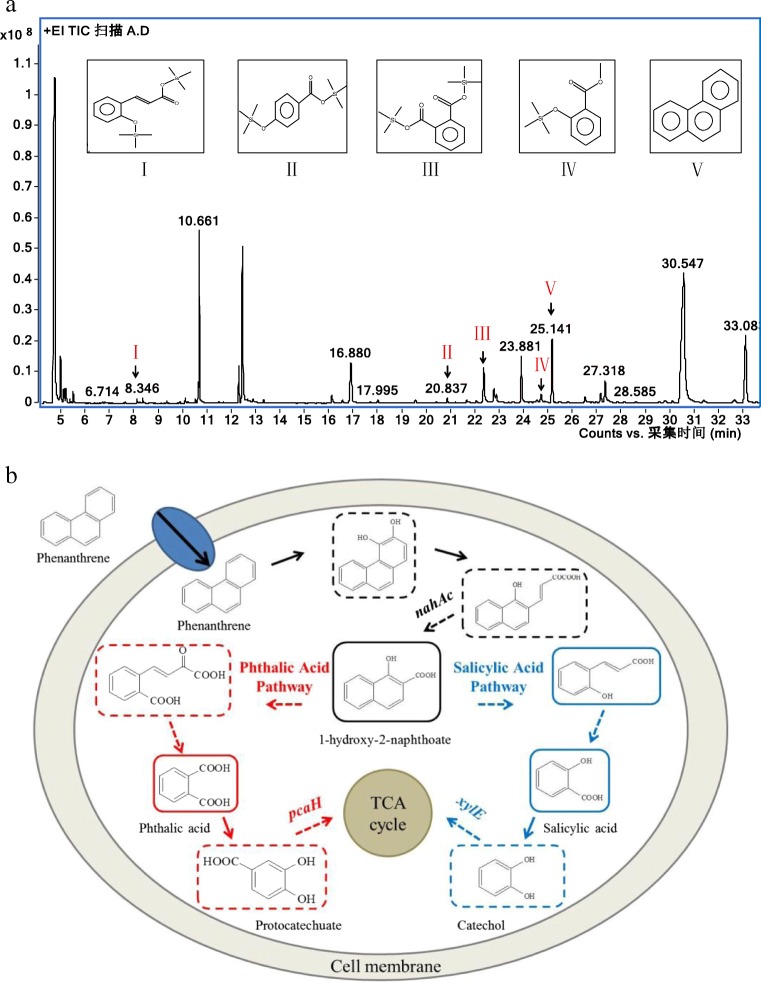
GC-MS profile of PHE-degrading culture (**a**) by *Pseudomonas* sp. strain DGYH-12 and main PHE degradation pathways in *Pseudomonas* sp. strain DGYH-12 (**b**) based on the detection of phthalic acid and salicylic acid. The intermediate metabolites shown in the solid line rectangle were detected; those shown in the dotted line rectangle were supposed to be detected based on the literature and database. The solid arrows indicate one step, and the dotted arrows indicate multiple steps

**Fig. 6:**
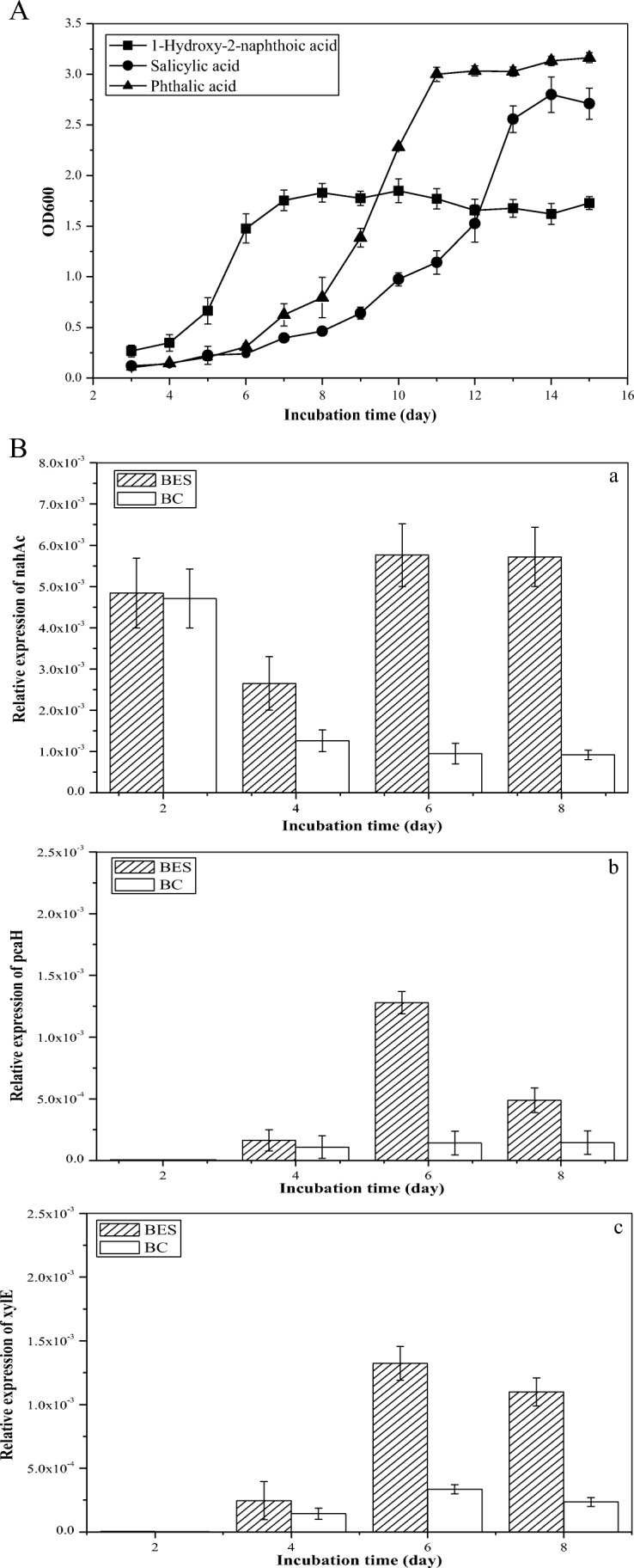
Growth of *Pseudomonas* sp. strain DGYH-12 upon utilization of 1-hydroxy-2-naphthoic acid (black square), salicylic acid (black circle), and phthalic acid (black triangle) as the sole carbon source (**A**) and relative expression of the genes (**B**) *nahAc* (*a*), *pcaH* (*b*), and *xylE* (*c*) in *Pseudomonas* sp. strain DGYH-12. The genes *nahAc*, *pcaH*, and *xylE* data were compared with different incubation times, respectively. The significant differences were marked with normal letters; the different normal letters on the bar indicate significant difference among settings at 0.05 level according to the Duncan test. The different letters in BES and BC data have no comparative relationship with each other

The metabolic pathway of strain DGYH-12 in PHE biodegradation based on the detected intermediate metabolites is shown in Fig. 5b. Among the metabolites, phthalic acid and salicylic acid were detected (Fig. 5a), which indicated that strain DGYH-12 has both a phthalic acid pathway and salicylic acid pathway in PHE degradation. Figure 6A demonstrates that strain DGYH-12 could grow well with 1-hydroxy-2-naphthoate, phthalic acid, and salicylic acid as the sole carbon source, which supports the results in Fig. 5b and further proves that strain DGYH-12 has two biodegradation pathways for PHE degradation. The two degradation pathways increase the complexity of metabolism and improve the adaptability in a complicated external environment. In addition, it could make strain DGYH-12 have higher PHE-degrading capabilities.

### Comparative expression of *nahAc* gene, *pcaH* gene, and *xylE* gene

To confirm that strain DGYH-12 has two degrading pathways and clarify how micro-electric stimulation affected the gene expression of the degradation enzymes involved in PHE biodegradation, RT-PCR reactions for *nahAc*, *pcaH*, *xylE*, and 16S rRNA transcripts were carried out on total RNA extracted from cells grown in the BES and a biological control upon PHE as the sole carbon source.

In Fig. 6B(a), the *nahAc* gene expression levels in the BES were 1.03, 2.10, 6.06, and 6.23 times higher than biological control at 2 days, 4 days, 6 days, and 8 days, respectively. Initially, the micro-electric field stimulation had little influence on the *nahAc* gene expression. The *nahAc* expression levels in both the BES and the biological control were relatively high at a 2-day incubation time, which could be induced by PHE. The gene *nahAc* was associated with the initial catalysis step during the PHE biodegradation process (Isaac et al. 2015); therefore, at day 2, the *nahAc* expressed more than the other PHE degradation gene (Fig. 6B(a)). From the day 2 to day 8, micro-electric stimulation significantly promoted the gene expression of *nahAc*.

In Fig. 6B(b), B(c), no gene expression of *pcaH* and *xylE* was detected at an incubation time of 2 days in both the BES and the biological control because the genes *pcaH* and *xylE* played important roles downstream of the phthalic acid pathway and salicylic acid pathway, respectively. Therefore, it was not the time for genes *pcaH* and *xylE* to be expressed. The *pcaH* gene expression levels in the BES were 1.49, 9.00, and 3.36 times higher than those in the biological control at 4 days, 6 days, and 8 days, respectively (Fig. 6B(b)). The gene *pcaH* was expressed in the phthalic acid metabolic pathway (Badejo et al. 2013) at days 4 and 6. The gene expression increased notably, and then, the growth curve of strain DGYH-12 in the phthalic acid degradation process began to rise at day 4 (Fig. 6A). The same situation occurred in the gene expression levels of *xylE* in the BES, which were 1.71, 3.93, and 4.66 times higher than those in the biological control at 4 days, 6 days, and 8 days, respectively (Fig. 6B(c)). *xylE* encoded the catechol-1, 2-dioxygenase, which is related to the salicylic acid metabolic pathway (Edlund and Jansson 2008). The salicylic acid degradation curve of the same change process is shown in Fig. 6A. From 4 to 6 days, the *pcaH* and *xylE* gene expression levels both increased dramatically, reached a peak level at 6 days, and then declined at 8 days.

Genes *pcaH* and *xylE* were successfully cloned in the total DNA of strain DGYH-12, which further illustrates that strain DGYH-12 had both a phthalic acid pathway and salicylic acid degradation pathway. On the whole, the gene expressions of both *pcaH* and *xylE* in the BES were significantly higher than the biological control, which proved that micro-electric stimulation could promote PHE degradation-related enzyme activity.

## Conclusions

1. This work is the first demonstration of how micro-electric stimulation promotes the performance of a PHE-degrading strain in detail and how micro-electric stimulation enhances the EPS production of *Pseudomonas* sp. DGYH-12.
2. In addition, it is also the first report to directly demonstrate that *Pseudomonas* sp. degrades PHE via both the phthalic acid and salicylic acid pathways. The strain showed stronger environmental adaptability and efficient PHE degradation activity.
3. These results are expected to offer a more detailed understanding on mechanism of the micro-electric stimulation influences PHE-degrading strain and offers a preliminary orientation for using BESs in PHE-contaminating wastewater treatments.

## Electronic supplementary material

ESM 1 (DOCX 277 kb)
