# Supplementary material for: Direct micro-electric stimulation alters phenanthrene-degrading metabolic activities of Pseudomonas sp. strain DGYH-12 in modified bioelectrochemical system
Source: Environ Sci Pollut Res Int. 2019 Sep 2;26(30):31449–62. doi: 10.1007/s11356-019-05670-5 (PMC6828628; doi:10.1007/s11356-019-05670-5)
Supplement: Supplementary file 1 — (DOCX 277 kb) [file 11356_2019_5670_MOESM1_ESM.docx]

**Supporting information**


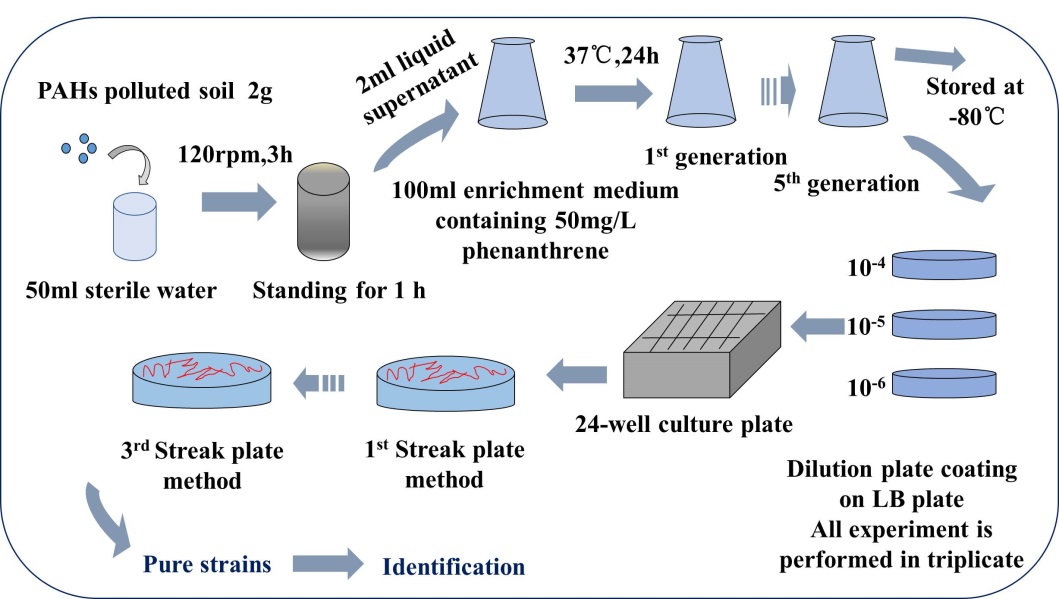


**Fig. 1** Isolation process of phenanthrene degrading bacteria

**
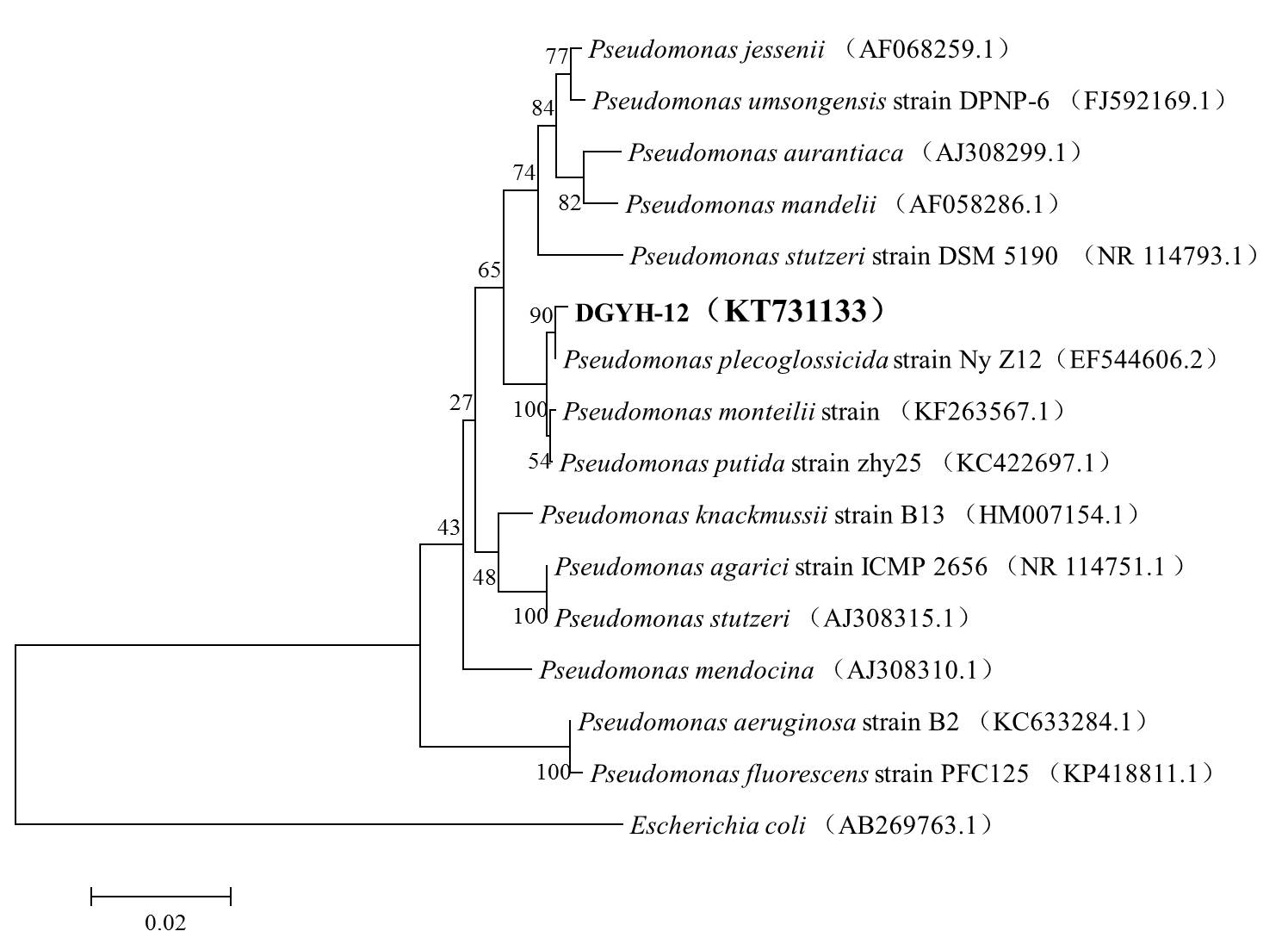
**

**Fig.2** Phylogenetic tree of *Pseudomonas plecoglossicida* strain DGYH-12

The scale bar, 0.02 Knuc unit. Number at the branch points indicate bootstrap values (%) based on a neighbor-joining analysis of 1000 replicate datasets. NCBI accession numbers are given in parentheses.

**Table1** Physiological and biochemical properties of *Pseudomonas* sp. DGYH-12

| **Number** | **Characteristic** | ***Pseudomonas plecoglossicida* strain DGYH-12** |
| --- | --- | --- |
| 1 | Lactose | **﹣** |
| 2 | Indole | **﹣** |
| 3 | Cellobiose | **﹣** |
| 4 | Xylose | **﹣** |
| 5 | Melibiose | **﹣** |
| 6 | Rhamnose | **﹣** |
| 7 | Saccharose | **﹣** |
| 8 | Raffinose | **﹣** |
| 9 | ONPG | **﹣** |
| 10 | Malonate | **﹣** |
| 11 | Urea | **+** |
| 12 | Lysine decarboxylase | **+** |
| 13 | Ornithine decarboxylase | **+** |
| 14 | Gelatin | **﹣** |
| 15 | Inositol | **﹣** |
| 16 | Sorbitol | **﹣** |
| 17 | Mannitol | **﹣** |
| 18 | Amygdalin | **﹣** |
| 19 | Esculoside | **﹣** |
| 20 | Melezitose | **﹣** |
| 21 | Xylitol | **﹣** |
| 22 | Salicin | **﹣** |

Note：+ means the strain DGYH-12 can utilize the substances, and – means the strain DGYH-12 cannot utilize the substances.
